# Supplementary material for: The role of P2Y12 in the kinetics of microglial self-renewal and maturation in the adult visual cortex in vivo
Source: eLife. 2021 Jul 12;10:e61173. doi: 10.7554/eLife.61173 (PMC8341987; doi:10.7554/eLife.61173)
Supplement: Figure 5—source code 1. [file elife-61173-fig5-code1.zip › Source Code Figure 5 Source Data File 1.docx]

Source code-Figure 5-Source Data File 1: Code to generate the mathematical model simulation

Source code-Figure 5-Source Data File 1: Mathematical Modeling of microglial repopulation. (A-C) Mathematical modeling of microglial repopulation using measured kinetic parameters. Empirical p values from the simulation are plotted for a minimum 4-hour delay (A) and 8-hour delay (B) between subsequent divisions; each animal is represented by a line (n=10).

doublet.sim <- function(n.cells, p.cells, obs.rates){

#Function takes three parameters

#n.cells is the starting number of cells in the population

#p.cells is the probability that a cell will be selected for division

#obs.rates is the vector of division rates that will be sampled from

#Number of cells at time window 0

n0 <- n.cells

#Number of cells selected for division

d0 <- rbinom(n = 1, size = n0, prob = p.cells)

#Sampled division rates for d0 cells.

samp0 <- sample(x = obs.rates, size = d0, replace = TRUE)

#Number of cells at time window 4

n4 <- n0 - d0 + 2*sum(samp0 == 4)

#Number of cells selected for division

d4 <- rbinom(n = 1, size = n4, prob = p.cells)

#Sampled division rates for d4 cells.

samp4 <- sample(x = obs.rates, size = d4, replace = TRUE)

#Number of cells at time window 8

n8 <- n4 - d4 + 2*(sum(samp0 == 8) +

sum(samp4 == 4))

#Number of cells selected for division

d8 <- rbinom(n = 1, size = n8, prob = p.cells)

#Sampled division rates for d8 cells

samp8 <- sample(x = obs.rates, size = d8, replace = TRUE)

#Number of cells at time window 12

n12 <- n8 - d8 + 2*(sum(samp0 == 12) +

sum(samp4 == 8) +

sum(samp8 == 4))

#Number of cells selected for division

d12 <- rbinom(n = 1, size = n12, prob = p.cells)

#Sampled division rates for d12 cells

samp12 <- sample(x = obs.rates, size = d12, replace = TRUE)

#Number of cells at time window 16

n16 <- n12 - d12 + 2*(sum(samp0 == 16) +

sum(samp4 == 12) +

sum(samp8 == 8) +

sum(samp12 == 4))

#Number of cells selected for division

d16 <- rbinom(n = 1, size = n16, prob = p.cells)

#Sampled division rates for d16 cells

samp16 <- sample(x = obs.rates, size = d16, replace = TRUE)

#Number of cells at time window 20

n20 <- n16 - d16 + 2*(sum(samp0 == 20) +

sum(samp4 == 16) +

sum(samp8 == 12) +

sum(samp12 == 8) +

sum(samp16 == 4))

#Number of cells selected for division

d20 <- rbinom(n = 1, size = n20, prob = p.cells)

#Sampled division rates for d20 cells

samp20 <- sample(x = obs.rates, size = d20, replace = TRUE)

#Number of cells that have divided at time window 24

n24 <- n20 - d20 + 2*(sum(samp0 == 24) +

sum(samp4 == 20) +

sum(samp8 == 16) +

sum(samp12 == 12) +

sum(samp16 == 8) +

sum(samp20 == 4))

#Total number of cells in the population after 24 hours. Includes n24 cells that have divided

#and all cells still in the process of division.

nFinal <- n24 + (sum(samp4 > 20) +

sum(samp8 > 16) +

sum(samp12 > 12) +

sum(samp16 > 8) +

sum(samp20 > 4))

print(nFinal)

}
